# Adult intussusception in the era of HIV/AIDS: A case report

**DOI:** 10.4102/sajid.v38i1.534

**Published:** 2023-08-28

**Authors:** Tshepang A. Motsepe, Thekganang A. Machete, Noluthando Ziqubu

**Affiliations:** 1 Department of General Surgery, School of Medicine, Sefako Makgatho Health Sciences University, Pretoria, South Africa; 2 Department of Nursing Sciences, Dr. George Mukhari Academic Hospital, Pretoria, South Africa

**Keywords:** non-Hodgkin’s lymphoma (NHL), intussusception, bowel obstruction, immunosuppression, lead point, anaemia, granuloma, intestinal lymphoma

## Abstract

The high incidence of HIV infection in South Africa has been associated with a proportional increase in AIDS-defining cancers, including non-Hodgkin’s lymphomas (NHL). Intussusception is a rare presentation of NHL, accounting for 1% – 5% of all cases of small bowel obstruction.

**Contribution:**

To our knowledge, this is the first reported case of B-cell NHL presenting with intussusceptions and small bowel obstruction in South Africa.

## Introduction

Sub-Saharan Africa records the highest HIV infections globally.^1^ The prevalence of HIV in South Africa is estimated at 20% for individuals aged 15–49 years,^2^ and this has led to it having the largest anti-retroviral drug programme in the world.^3^ HIV has many associated complications, including malignancies. The incidence of AIDS-defining cancers, known to be Kaposi sarcoma, non-Hodgkin’s lymphoma (NHL) and cervical cancer, is much higher in individuals living with HIV than in the general population.^4^ The incidence of NHL in the general population is estimated at 18.6 of 100 000 individuals^5^ while in people living with HIV the incidence rates of 205 out of 100 000 persons for those on combination antiretroviral therapy (cART) and 463 out of 100 000 persons for those not on cART.^6^

Non-Hodgkin’s lymphoma is the second most common neoplasm in people living with HIV. Diffuse large B-cell NHL is the most usual histological type.^7^ The gut is the most common extranodal site affected by lymphoma. Gastrointestinal lymphoma is usually because of secondary metastatic nodal disease affecting the stomach, small bowel and colon.^7^ The ileum is the most common site affected by small intestinal lymphoma, followed by the jejunum and duodenum.^8^ Intussusception is a rare presentation of NHL and is often seen in the case of a diffuse large B-cell NHL.^8^ Adult intussusception represents 5% of all intussusception cases and 1% – 5% of all cases of bowel obstruction.^8,9^

## Case report

A 23-year-old female presented with features of small bowel obstruction. She complained of vomiting for the past 3 days, failure to pass stools for 5 days and abdominal distension. She was diagnosed with HIV four years earlier, and had a CD4 count of 294 x 10^6^ cells/µL with an undetectable viral load at presentation. Her cART comprised daily fixed combination of tenofovir, emtricitabine and efavirenz for the past four years. She reported no fever, weight loss or night sweats. On physical examination, she had an elevated blood pressure of 156/106 mmHg and a tachycardia of 150 beats per minute; however, she was apyrexial with a normal respiratory rate. Her abdomen was markedly distended, firm and generally tender. She had involuntary guarding and rebound tenderness. Digital rectal examination revealed an empty rectum. Per vaginal examination and assessment of other systems were unremarkable.

The patient’s biochemistry results are shown in Table 1. Patient’s elevated white cell count (WCC) and inflammatory marker C-reactive protein (CRP) as well as neutrophilia indicated a systemic infection. Haemoglobin level was also abnormally low at 4.7 g/dL. All other parameters, including renal and liver function tests as well as arterial blood gas analysis (ABG), were normal.

**TABLE 1:** The patient’s laboratory results.

Blood investigations	Patient’s results	Normal ranges†
White cell count	21.50 × 10^9^/L	3.90 – 12.60 × 10^9^/L
Haemoglobin	4.7 g/dL	11.6 g/dL – 16.4 g/dL
Platelet count	360 × 10^9^/L	12.4 – 17.3 × 10^9^/L
Neutrophils	19.8 × 10^9^/L	1.6 – 8.30 × 10^9^/L
Lymphocytes	0.69 × 10^9^/L	1.40 – 4.50 × 10^9^/L
Monocytes	0.75 × 10^9^/L	0.20 – 0.080 × 10^9^/L
Eosinophils	0.04 × 10^9^/L	0.00 – 0.40 × 10^9^/L
C-reactive protein (CRP)	308 mg/L	˂ 10 mg/L

† , Reference ranges for laboratory values are from the South African National Health Laboratory Service (NHLS).

**FIGURE 1:**
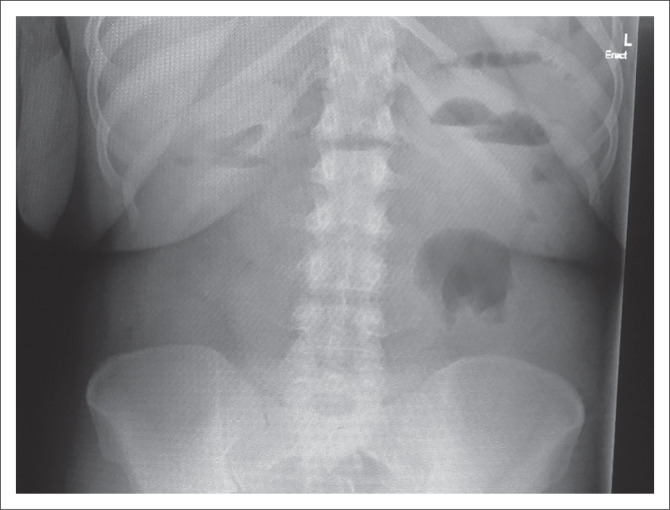
The patient’s erect abdominal radiograph. Multiple air-fluid levels are depicted.

**FIGURE 2:**
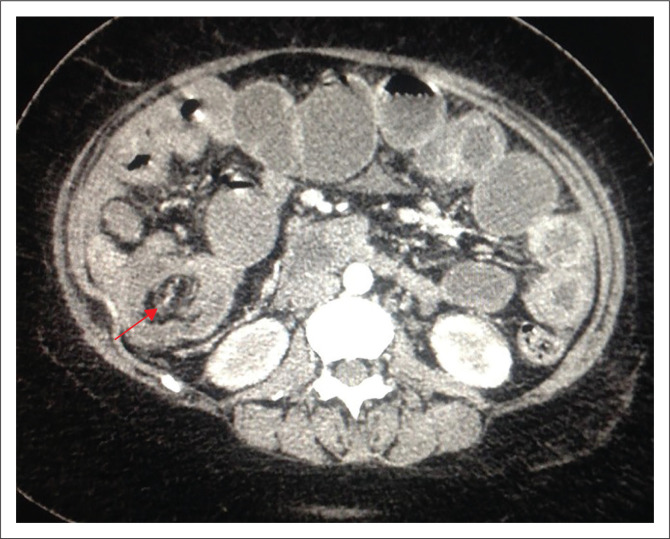
The patient’s computed tomography (CT) image (axial view) in the arterial phase. The red arrow indicates a target lesion at the ileo-caecal junction. Dilated loops of small bowel are seen.

Air fluid levels were demonstrable on erect abdominal X-rays (see Figure 1). Computed tomography (CT) scan showed target signs with small bowel wall thickening and dilation. Intra-abdominal fluid collection was also evident (see Figure 2). The following differential diagnoses were considered: Abdominal tuberculosis, lymphomas, small bowel neuroendocrine tumours, gastrointestinal stromal tumours (GIST) and sarcomas. The radiological findings suggested intussusception, complicated by small bowel obstruction.

**FIGURE 3:**
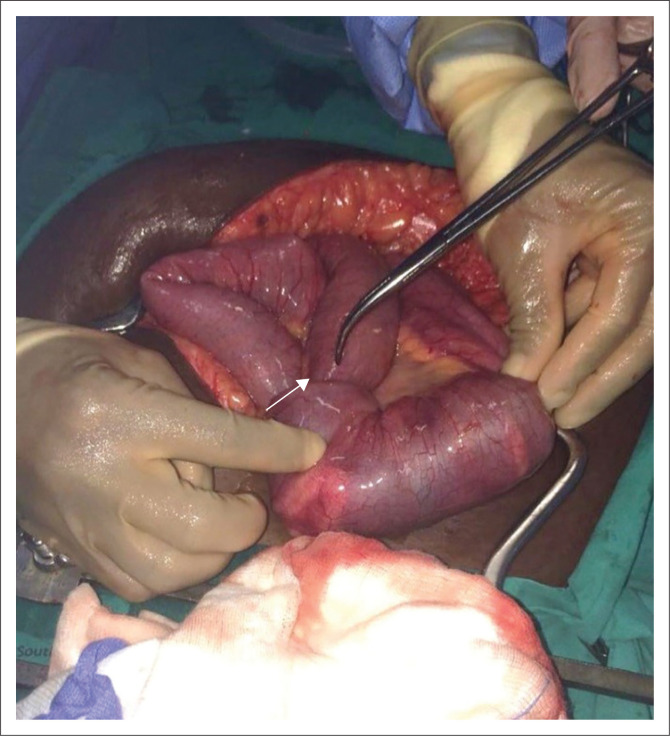
The patient’s image showing (white arrow) a mid-jejunal intussusception. A perforated intramural granuloma is visible.

After fluid resuscitation, the patient was prepared for an exploratory laparotomy. Intraoperative findings included a reducible intussusception at the ileo-caecal juncture and another at the mid-jejunum, which needed a wedge resection and a jejunostomy (see Figure 3). Bilateral ovarian tumours were observed, with the left larger than the right. Multiple intramural granulomatous masses, measuring approximately 1.5 cm × 1.5 cm, were disseminated over the entire small bowel. Some granulomas in the small bowel had broken down to release pus. The small bowel looked oedematous and had areas of haematoma. Jejunal intussusception and left ovary specimens were sent for histological analysis. A high-grade large B-cell NHL was found in the two specimens. The patient fully recovered from the bowel obstruction and gained full functional health while awaiting chemotherapy.

## Discussion

The clinical manifestations of primary intestinal lymphoma include nausea and vomiting, insidious abdominal pains, ‘B’ symptoms (i.e., weight loss, fever and night sweats), abdominal mass and haematochesia.^10^ However, our patient presented late with features of small bowel obstruction and peritonism. Peritoneal signs and elevated septic markers were probably attributed to ruptured granulomas with intra-abdominal pus. The patient had anaemia with the haemoglobin of 4.7 g/dL. During the post-operative period in the ward, bone marrow investigation was performed and confirmed anaemia of chronic disease and tumour infiltration.

Lymphomas are a diverse spectrum of neoplastic disorders that originate from either T- or B-lymphocytes. Broadly speaking, they are divided into Hodgkin’s and non-Hodgkin’s lymphomas.^11^ Diffuse large B-cell NHL is the commonest HIV-associated lymphoma.^12^ The development of HIV-associated lymphomas includes an interaction of different biologic variables, such as chronic antigen stimulation, co-infection with oncogenic viruses, genetic abnormalities and cytokine dysregulation, which together influence the likelihood of developing a specific type of lymphoma.^12^

Bowel obstruction in the context of HIV poses a unique challenge to the treating physician who must contemplate the possibility of opportunistic infections and malignancies, which may not be prevalent in the general population.^13^ Non-Hodgkin’s lymphoma and Kaposi’s sarcoma may lead to intestinal obstruction from an intraluminal space occupying mass or by acting as a lead point for intussusception.^13^ Abdominal mycobacterial infection can cause obstruction by fibrotic adhesions due to terminal ileitis.^13^ Cytomegalovirus^14^ and cryptococcal small bowel obstruction^15^ have also been described. However, other causes of obstruction such as post-surgery small bowel adhesions and herniae should still be considered.^13^ In this case our patient presented with with two sites of intussusceptions with NHL granulomas acting as lead points.

Intussusception occurs when a more proximal segment of bowel (intussusceptum) folds into the lumen of an adjacent distal segment of another bowel (intussuscipiens).^16^ This is postulated to be because of deranged bowel peristalsis at the site of the lesion, which forms a lead point for the intussusceptum.^16^ A lead point may be a tumour, polyp, Meckel diverticulum or even an appendix stump.^16^ In patients with no obvious identifiable lead point, intussusception may be from submucosal bowel oedema, fibrous adhesions or irregular intestinal contractions.^16^

While abdominal ultrasound is considered comparable to CT scan for sensitivity and specificity, it is operator dependent.^10^ It carries the advantage of being relatively cheap, non-invasive, and readily available.^10^ The classic ultrasonographic features of intussusception include target sign or doughnut sign on axial view as well as pseudo-kidney sign or hayfork sign on longitudinal plane.^10^ Computed tomography scan has a superior value in defining the location and in characterising the tumour and its association with surrounding structures.^10^ It is a modality of choice for staging the disease.^10^ Surgery is indicated for the management of intussusception and/or bowel obstruction.^10^ An emergency laparotomy was performed as the patient in the case presented acutely with a complicated small bowel obstruction. Surgery also offers the opportunity to biopsy the lesion for diagnostic purposes.^10^ The gold standard of treatment of lymphoma is chemotherapy, while surgery plays a role in the management of associated complications, such as bowel obstruction.^10^ The use of the regimen cyclophosphamide, doxorubicin, vincristine and prednisone (CHOP) is warranted in both immunosuppressed and immunocompetent patients.^10^

## Conclusion

HIV is a major cause of morbidity and mortality in South Africa. However, widespread cART availability has reduced the rate of new transmission and complications of HIV. The case highlights how HIV-related lymphomatous granulomas, acting as lead points, may cause intussusception and subsequent bowel obstruction.

The presentation of intussusception from granulomatous lesions of lymphoma is uncommon with few reported cases internationally. To our knowledge, this is the first reported case in South Africa of B-cell NHL presenting with two sites of intussusceptions that are located at the ileo-caecal junction and the mid-jejunum. The incidence of bowel obstruction caused by intussusception in people with HIV is undetermined in South Africa. Therefore, this case report serves as a foundation for further research on the topic.
